# Alteration of the exDNA profile in blood serum of LLC-bearing mice under the decrease of tumour invasion potential by bovine pancreatic DNase I treatment

**DOI:** 10.1371/journal.pone.0171988

**Published:** 2017-02-21

**Authors:** Ludmila A. Alekseeva, Nadezhda L. Mironova, Evgenyi V. Brenner, Alexander M. Kurilshikov, Olga A. Patutina, Marina A. Zenkova

**Affiliations:** Institute of Chemical Biology and Fundamental Medicine, SB RAS, Novosibirsk, Russia; University of South Alabama Mitchell Cancer Institute, UNITED STATES

## Abstract

Taking into account recently obtained data indicating the participation of circulating extracellular DNA (exDNA) in tumorigenesis, enzymes with deoxyribonucleic activity have again been considered as potential antitumour and antimetastatic drugs. Previously, using murine Lewis lung carcinoma and hepatocellular carcinoma A1 tumour models, we have shown the antimetastatic activity of bovine DNase I, which correlates with an increase of DNase activity and a decrease of exDNA concentration in the blood serum of tumour-bearing mice. In this work, using next-generation sequencing on the ABS SOLiD^™^ 5.500 platform, we performed a search for molecular targets of DNase I by comparing the exDNA profiles of healthy animals, untreated animals with Lewis lung carcinoma (LLC) and those with LLC treated with DNase I. We found that upon DNase I treatment of LLC-bearing mice, together with inhibition of metastasis, a number of strong alterations in the patterns of exDNA were observed. The major differences in exDNA profiles between groups were: i) the level of GC-poor sequences increased during tumour development was reduced to that of healthy mice; ii) levels of sequences corresponding to tumour-associated genes *Hmga2*, *Myc* and *Jun* were reduced in the DNase I-treated group in comparison with non-treated mice; iii) 224 types of tandem repeat over-presented in untreated LLC-bearing mice were significantly reduced after DNase I treatment. The most important result obtained in the work is that DNase I decreased the level of B-subfamily repeats having homology to human ALU repeats, known as markers of carcinogenesis, to the level of healthy animals. Thus, the obtained data lead us to suppose that circulating exDNA plays a role in tumour dissemination, and alteration of multiple molecular targets in the bloodstream by DNase I reduces the invasive potential of tumours.

## Introduction

Extracellular DNA (exDNA) is a recently discovered component of blood plasma and its elevated level is a characteristic feature of patients with oncological diseases often associated with heavy tumor progression and poor prognosis [1–3]. It has been firmly established that circulating exDNA contains oncogenes including hypermethylated tumour suppressor genes, aberrant microsatellites, aberrant DNA methylation genes and rearranged chromosomes [4–7]. In this regard, diagnostic and prognostic tools are being developed based on determination of the total concentration of exDNA [8], the ratio of the levels of normal and mutant exDNA [9] and the incidence of certain types of aberrant exDNA[10], tandem repeats, etc. [11,12] in the blood of oncological patients. Today, most researchers agree that concentrations of exDNA could be used as a tool for early diagnosis of cancer in combination with other cancer markers [8].

Careful investigation of exDNA concentrations, components, patterns, etc. has become of use after the opening of its possible role in carcinogenesis that is supported by some hypotheses. One of them, the genometastatic hypothesis, has been proposed to describe the phenomenon of horizontal transfer of tumour-specific circulating exDNA originating from primary tumour cells into the healthy cells of distant organs [13–15]. Some authors hypothesize that oncogene-containing exDNA may behave like oncoviruses, and represents an alternative pathway for cancer metastasis. In other words, cancer has the propensity to settle down metastatically in specific tissues since there are DNA-binding proteins or receptors on the surface of these cells [16–23].

Taking into account the possible role of exDNA in tumour dissemination, two enzymes capable of destroying DNA have attracted the attention of researchers as antitumour drugs: bovine pancreatic DNase I [24] and human recombinant DNase I (dornase alfa) [25]. Using several tumour cell lines (Calu-1, SK-MES-1, HeLa, HEP-2 and L-929) it was shown that bovine pancreatic DNase I decreased the rate of tumour cell proliferation and reduced the exDNA concentration in culture medium [26]. The antimetastatic potential of bovine pancreatic DNase I has been demonstrated in vivo in models of spontaneous liver cancer and lymphocytic leukaemia (L5178Y-ML) [27–30]. In an orthotopic pancreatic cancer mouse model, Wen and colleagues showed that DNase I decreased the rate of metastasis development in vivo and reduced the migration and invasion potential of tumour cells in vitro, but had no effect on the migration of normal pancreatic ductal epithelial cells [31]. In addition, some attempts to use DNase I as a drug for the treatment of patients with various metastatic cancers have been made in several clinical trials [32,33]. However, despite this progress, information about the antitumour activity of DNase I and mechanisms mediating this activity remain fragmentary and unclear.

Previously, we have demonstrated the high antimetastatic activity of bovine pancreatic DNase I using two murine tumour models, Lewis lung carcinoma (LLC) and hepatocellular carcinoma A1 (HA-1) [34]. It was shown that, together with a decrease of metastasis number and area, DNase I treatment resulted in elevation of deoxyribonuclease activity in the blood plasma of tumour-bearing mice to the level of healthy animals following a reduction of abnormally enhanced concentration of blood serum exDNA.

In this study, we performed a search for molecular targets of DNase I among exDNA in the blood plasma of animals with LLC. For these purposes, we compared the exDNA profiles of healthy mice and mice with LLC, treated and not treated with DNase I. Accordingly, we constructed three DNA libraries of exDNA from the blood serum of healthy C57Bl/6J mice, LLC-bearing mice (control) and those treated with DNase I, and carried out analysis of these libraries by next-generation sequencing (NGS) on an ABS SOLiD^™^ 5.500 platform.

## Materials and methods

### Tumour strain

The LLC tumour strain was obtained from the vivarium at the Federal Research Centre (FRC) Institute of Cytology and Genetics (ICG) of the Siberian Branch of the Russian Academy of Sciences (SB RAS), Novosibirsk, Russia.

### Mice

Male 10–14-week-old C57Bl/6J (hereinafter, C57Bl/6) mice were obtained from the animal breeding facility within the FRC Institute of Cytology and Genetics of SB RAS (ICG of SB RAS), Novosibirsk, Russia. Mice were housed in plastic cages (ten animals per cage) under normal daylight conditions. Water and food were provided ad libitum. All animal procedures were carried out in strict accordance with the approved protocol and recommendations for proper use and care of laboratory animals (ECC Directive 2010/63/EU). The protocol and this study were specifically approved by the Committee on the Ethics of Animal Experiments of the Administration of the Siberian Branch of the Russian Academy of Sciences (permit number: 5-06-2015). Mice were euthanized by cervical dislocation under light ether narcosis and all efforts were made to minimize suffering.

At the start of the experiments, animal weight (mean ± SD) was 20.2 ± 1.5 g. To perform LLC studies, ten animals per group were used. The number of animals in each group was chosen in accordance with the requirement to provide statistically significant data.

### Tumour transplantation and design of animal experiments

Ten- to 14-week-old male C57Bl/6 mice were administered intramuscularly (i.m.) with LLC cells (10^6^ cells/mouse) suspended in 0.1 ml of saline buffer into the right thighs. On day 4 after tumour transplantation, each mouse with LLC was assigned to one of two groups (n = 10): 1, control, received i.m. saline buffer (0.1 ml); 2, received i.m. bovine pancreatic DNase I (Sigma, USA) at a dose of 0.12 mg/kg (0.1 ml). DNase I was administered daily except for weekends. The total number of injections was ten. Tumor size was determined every other day using caliper measurements in three perpendicular dimensions. Tumor volumes were calculated as V = (π/6 × length × width × height).

On day 15 after tumour transplantation, blood samples (0.2 ml) were collected from the retro-orbital sinus 60 min after the last DNase I injection. Then, mice were euthanized and the lungs were isolated and fixed in 4% neutral-buffered formaldehyde for 2 weeks.

Surface metastases in the lungs were counted using a binocular microscope. Blood samples from healthy mice (0.2 ml) were collected from the retro-orbital sinus two times with an interval of 7 days.

### Blood serum preparation

Blood serum was prepared from the whole blood of healthy animals and animals with LLC treated with saline buffer or DNase I by clot formation at 37°C for 30 min and at 4°C overnight, followed by clot discard and centrifugation (4000 rpm, 4°C, 20 min) to remove cell debris. Serum samples were stored at −70°C until use.

### Analysis of the number and area of metastases

For morphometric analysis, lungs were fixed in 10% neutral-buffered formalin (pH 7.0), routinely processed and embedded in paraffin. Paraffin sections (5 μm) were stained with haematoxylin and eosin, microscopically examined and scanned. Paraffin sections (5 μm) were stained with haematoxylin and eosin, microscopically examined and scanned (S1 Fig). Images were obtained using an Axiostar plus microscope equipped with an Axiocam MRc5 digital camera (Zeiss, Germany). The percentages of the internal metastases area were determined relative to the total area of sections using Adobe Photoshop Software. Each studied group included six mice and five random sections were studied in each specimen, as a result forming in total 30 fields for each group of mice.

Inhibition of metastases development was assessed by morphometry using the metastasis inhibition index (MII). The metastasis inhibition index (MII) was calculated as MII = [(mean metastasis area_control_ − mean metastasis area_experiment_)/mean metastasis area_control_] × 100%. The MII of the control group corresponds to 0%; MII = 100% means the absence of metastases.

The MII of the control group was taken as 0% and the MII, corresponding to 100%, reflected the absence of metastases.

### Selection for blood samples destined for exDNA isolation

At the end of the experiment after the number of metastases was calculated these data were analyzed and distribution curve was built. Taking into account that the distribution was abnormal, the outliners (extreme values) were discarded and as a result the distribution was brought to a normal. The samples from mice, forming normal distribution, were included in further analysis.

### Isolation of exDNA from blood serum

Serum samples of six animals of each group were pooled according to the groups. exDNA was isolated from the blood serum by extraction with phenol (phenol–Tris–HCl, pH 8.0) and chloroform, followed by concentration using a QIAquick^®^ Gel Extraction Kit (Qiagen, USA). The concentration of exDNA was measured by a Qubit^™^ fluorometer (Invitrogen, USA) using a Quant-iT^™^ dsDNA HS Assay Kit (Invitrogen, USA) according to the manufacturer’s recommendations.

### DNA library preparation and sequencing

Libraries of exDNA were prepared using a modified ABS SOLiD^™^ Fragment Library Construction Kit (Applied Biosystems^™^, USA). exDNA (~200 ng, Table 1) was sonicated using a Covaris S2 System for 155 s. After blunt-ending, exDNA fragments were ligated with double-stranded adapters by T4 ligase. The ligation products were then size-selected (150–250 bp) using 1.5% LE-agarose gel (Sigma, USA), and eluted from the gel using a QIAquick^®^ Gel Extraction Kit (Qiagen, USA). Further nick translation was performed (72°C, 20 min denaturation; −95°C, 5 min). Obtained libraries were amplified using primers specific for the adapters: 95°C, 15 s; 62°C, 15 s; 70°C, 4 min; six cycles. The obtained PCR products were size-selected (150–250 bp) using 1.5% LE-agarose gel, and eluted from the gel as described above. Templated bead preparation, emulsion PCR and deposition were performed following the standard ABS SOLiD^™^ 5.500 protocols. Three libraries, L_h_ (exDNA from blood serum of healthy mice), L_LLC_ (exDNA from blood serum of mice with LLC treated with saline buffer) and L_D_ (exDNA from blood serum of mice with LLC treated with DNase I) were obtained and sequenced using an ABS SOLiD^™^ 5.500 platform (the length of the reading fragment was 75 nucleotides). The total volume of the primary sequencing data was not less than 50 million sequences.

**Table 1:** Concentration of exDNA and DNA libraries at different stages of construction.

DNA- library design stage	Library type		
	L_h_^1^	L_LLC_^1^	L_D_^1^
^2^Serum exDNA concentration,ng/ml	77 ± 2	162 ± 5	110 ± 4
Initial quantity DNA for library preparation, ng	230	244	220
The amount of DNA libraries, ng	31.8	54.8	10.2

^1^L_h_, L_D_ and L_LLC_ are DNA libraries prepared from exDNA isolated from blood serum of healthy mice C57Bl/6J, mice with LLC treated with saline buffer and mice with LLC treated with DNase I at the dose of 0.12 mg/kg.

^2^Data on serum exDNA concentration were statistically analyzed using the Student’s t-test (two-tailed, unpaired). Data are presented as mean ± SEM. Data has statistically significant differences with *p < 10*^*−12*^ (L_h_ *vs* L_LLC_), *p < 10*^*−7*^ (L_D_ *vs* L_LLC_) and *p < 10*^*−7*^ (L_h_ *vs* L_D_).

### Sequencing data analysis

Sequencing data have been submitted to the NCBI BioProject (accession number PRJNA313482).

The sequencing data were pre-processed with the Trimmomatic 0.32 tool [35] to remove adapters and low-quality sequences. After barcode trimming, we assessed the quality of the sequencing data using FastQC software. The data were mapped to the reference comprising 980 repeat sequences presented in the *Mus musculus* genome. Bioscope software version 1.3 was used for mapping. Read counts per repeat family were normalized by library size and then log-transformed. To estimate tumour-associated gene coverage, the data was mapped to the *Mus musculus* genome (MM9) with Bioscope 1.3. The number of reads mapped to the exons of *Hmga2*, *Fos*, *Myc* and *Jun* was standardized to the library size as RPM (number of reads per million reads in library).

### Real-time RT-PCR

The alteration in exDNA profile of C-Myc was detected by SYBR-Green-based real-time RT-PCR. The PCR mixture (20 μl) consisted of 5 μl of exDNA (0.1–0.5 ng per reaction), 10 μl of SYBR–Green-containing BioMasterCor HS-qPCR (BiolabMix, Russia) and 0.4 μmol of each primer. The following primers were used: Myc sense 5’-CGACTACGACTCCGTACAGC-3’, Myc antisense 5’-CCAGATATCCTCACTGGGCG-3’; β-actin sense 5’-TCTTTGCAGCTCCTTCGTTG -3’, β-actin antisense 5’-AGTGAGGTACTAGCCACGAGA-3’. The cycling conditions were 95°C for 6 min for pre-denaturation; and 95°C for 15 s, 59°C for 15 s, 65°C for 45 s, for 40 cycles, followed by melting curves analysis. The expression level of each gene was indicated by the number of cycles needed for the DNA amplification to reach a threshold. The amount of DNA was calculated from the number of cycles by using standard curves and the results were normalized to β-actin.

### Statistics

The animal data were statistically processed using one-way ANOVA. Post-hoc testing was completed using Fisher’s least significant differences (LSD); p <0.05 was considered to be statistically significant. The statistical package STATISTICA version 10.0 was used for analysis. The data were statistically processed using Student’s t-test; p < 0.05 was considered to be statistically significant.

## Results

### Experimental scheme

Previously, we demonstrated the antimetastatic activity of bovine pancreatic DNase I using two tumour models, LLC and HA-1 [34]. It was shown that DNase I in the dose range 0.02–2.3 mg/kg inhibited the development of metastasis by 60–90% and caused an increase of the deoxyribonucleic activity of blood plasma and a reduction of the concentration of circulating exDNA.

**Fig 1:**
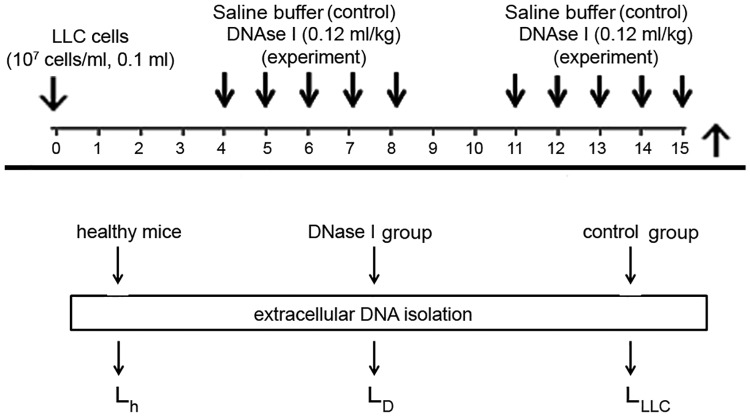
Scheme of the animal experiment and library preparation. LLC cells (10^6^ cells, 0.1 ml) were transplanted intramuscularly (i.m.) into C57Bl/6J mice (male). Starting from day 4 after the tumour transplantation, the animals received i.m. saline buffer or DNase I (0.12 mg/kg) for 10 days. At day 15, 1 h after the last injection, blood sampling was performed, then animals were euthanized and the lungs were taken. ExDNA was isolated from blood serum, and DNA libraries were prepared for subsequent sequencing.

To find possible molecular targets of DNase I among circulating exDNA, we analysed the profiles of exDNA from the blood of mice with LLC after treatment with DNase I using an NGS technique. The experimental scheme is shown in Fig 1. LLC cells (10^6^ cells) were transplanted i.m. into the right thigh muscle of C57Bl/6J mice and, starting from the fourth day (the beginning of the exponential growth of the tumour and metastasis [34]), mice received i.m. saline buffer (control) or DNase I (0.12 mg /kg) for 2 weeks. The DNase I dose of 0.12 mg/kg, providing a high antimetastatic effect in the LLC model, was chosen earlier [34].

DNA sampling was performed at the end of experiment (day 15) because at this stage of tumour development, sharp differences in tumour progression between experimental (DNase I treatment) and control (saline buffer) samples were observed [34]. Moreover, we believe that at this time point of the experiment, all alterations and differences between the groups were in a steady state.

### Evaluation of antimetastatic activity of DNase I and sampling for analysis of exDNA

**Fig 2:**
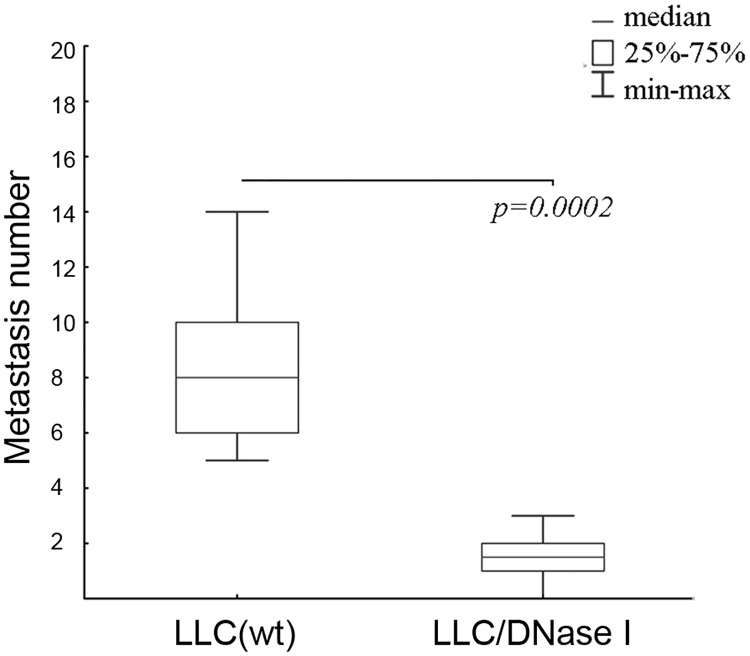
Number of metastases in lungs of mice with LLC treated with saline buffer (LLC(wt)) or DNase I (LLC/DNase I). LLC cells were injected intramuscularly (i.m.) into C57Bl/6J mice (n = 10). On day 4 after LLC cells inoculation the mice were daily intramuscularly treated with DNase I (0.12 mg/kg). Data are presented as mean ± SEM. Statistical significance is p < 0.0002.

Since in these experiments blood sampling, exDNA isolation and DNA library construction were intended to reveal factors associated with tumour progression and metastasis spreading, prior to DNA isolation the number and area of metastases in the lungs of mice with LLC treated with DNase I were evaluated. Microscopic examination of the lung surface revealed that treatment with DNase I resulted in a significant decrease in the number of metastases (Fig 2). The average number of metastases in the control group was 8 ± 2, whereas the number of metastases in the group treated with DNase I was 2 ± 1, (p < 0.05). Morphometric analysis of lungs revealed that MII, showing a reduction of the area occupied by metastasis, was 36.5% (p < 0.05). No effect of DNase I on the primary tumor size was observed. For further analysis, blood serum from the six mice enclosed in normal distribution after outliners exclusion was pooled and used for L_D_ DNA library preparation. Accordingly, blood samples of the six mice from the control group showing the most aggressive tumour progression were used to create the DNA library L_LLC_.

### Construction of DNA libraries

ExDNA was isolated from blood as described in the Materials and Methods section, and measurements of the concentration of exDNA in the pooled samples showed that in the blood of healthy animals, the exDNA level was low (77 ng/ml) and tumour development was characterized by a two-fold increase in the exDNA level (162 ng/ml), while DNase I treatment led to a decrease in the exDNA level (110 ng/ml), closer to healthy animals than to the control group (Table 1).

DNA libraries were prepared as described in the Materials and Methods section according to an ABS SOLiD^™^ Fragment Library Construction Kit with some modifications. This sequencing technique allows fragments of 50 bp to be sequenced with maximum efficiency. The first modification was sonication of exDNA samples to unify the length of the fragments to 50 bp, as exDNA samples were characterized by a significant amount of 150–200 bp fragments, a two- to three-fold lower amount of 400–10000 bp fragments and an absence of fragments shorter than 150 bp. The second modification of the DNA library construction protocol was a reduction of the number of PCR cycles to six, which allowed, on the one hand, avoidance of over-representation of fragments and, on the other hand, enough material to be obtained for sequencing start-up.

After the second size-selection of 150–250 bp products, the following libraries were obtained: L_h1_ and L_h2_, representing exDNA of healthy mice (two technical replicates); L_LLC_−exDNA of LLC-bearing mice, receiving saline buffer and L_D_−exDNA of mice with LLC, treated with DNase I. Amounts of ready-to-sequence DNA libraries were 31.8, 54.8 and 10.2 ng for L_h_, L_LLC_ and L_D_, respectively (Table 1).

### Analysis of sequencing data

On average, ~25–67 million single-end reads were obtained for each sample of exDNA by means of SOLiD non-stranded sequencing. The sequencing data were pre-processed with the Trimmomatic 0.32 tool to remove adapters and low-quality sequences. After barcode trimming, we assessed the quality of the sequencing data using FastQC software and mapped them onto the *Mus musculus* reference genome assembly MM9 (Ensemble release 67) using Bowtie version 1.

**Fig 3:**
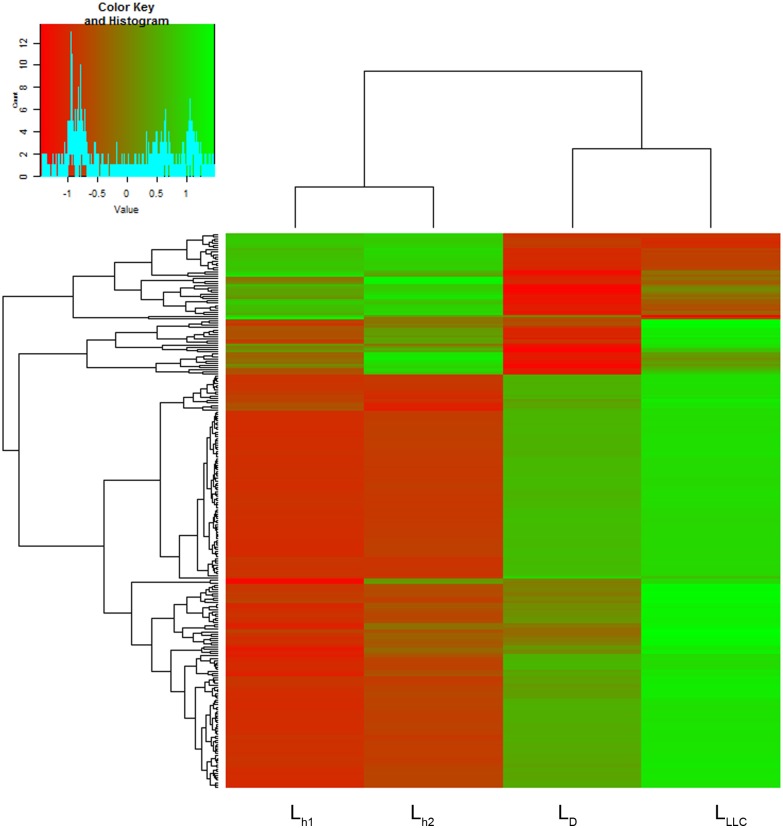
Heatmap on the abundance of tandem repeats in DNA libraries prepared using exDNA from blood serum of healthy mice (L_h1_ and L_h2_), mice with LLC treated with saline buffer (L_LLC_) and mice with LLC treated with DNase I (L_D_). The decreased and increased tandem repeats are indicated by range of red and green intensities, respectively. Dendrograms were derived by complete linkage clustering of tandem repeats or samples using Euclidean distances between scaled log-transformed RPKM values (Colour Key), respectively.

**Fig 4:**
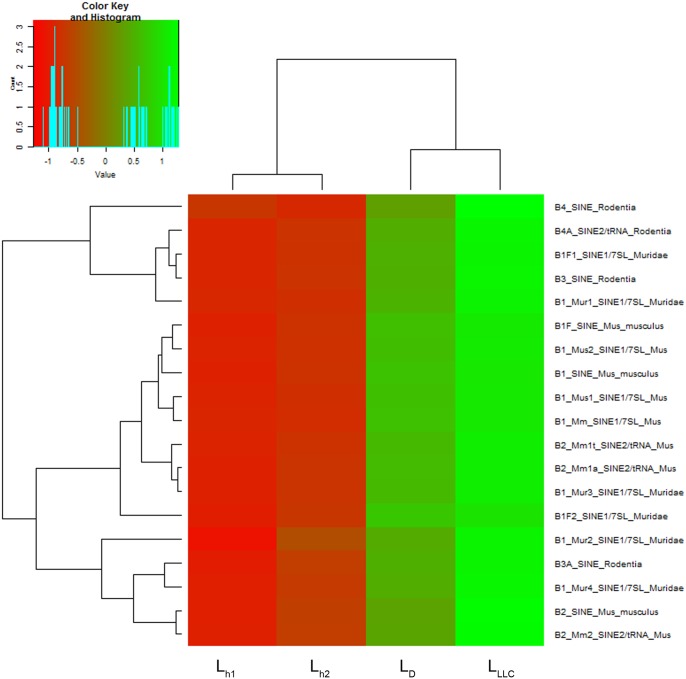
Heatmap on the abundance of B-family tandem repeats homologous to human ALU repeats in DNA libraries L_h1_, L_h2_, L_LLC_ and L_D_. The decreased and increased tandem repeats are indicated by range of red and green intensities, respectively. Dendrograms were derived by complete linkage clustering of tandem repeats or samples using Euclidean distances between scaled log-transformed RPKM values (Colour Key), respectively.

**Fig 5:**
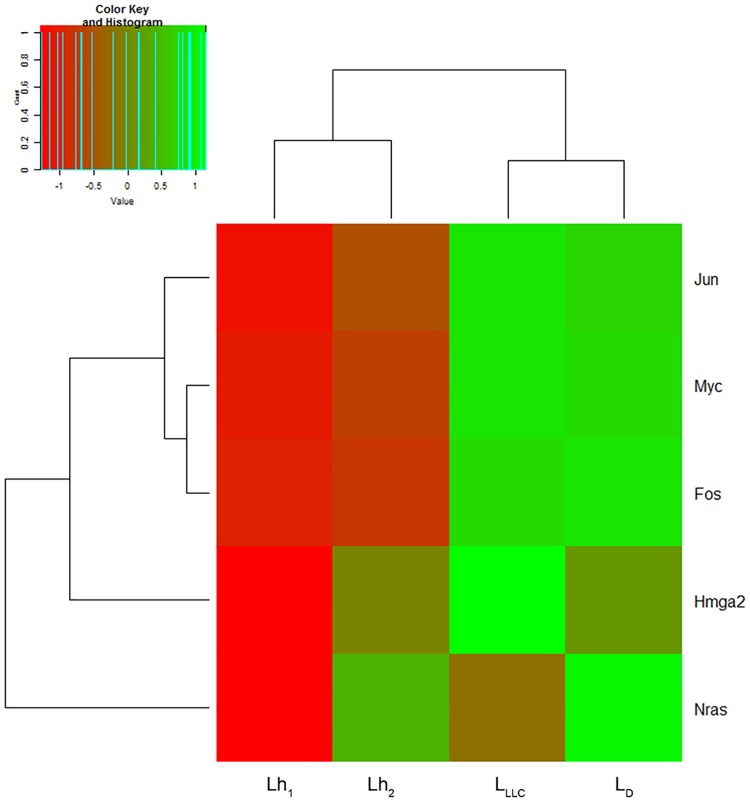
Heatmap on the abundance of fragments encoding tumour-associated genes *Hmga2*, *Fos*, *Myc*, *Nras* and *Jun* in DNA libraries L_h1_, L_h2_, L_LLC_ and L_D_. The decreased and increased genes are indicated by range of red and green intensities, respectively. Dendrograms were derived by complete linkage clustering of tumour-associated genes or samples using Euclidean distances between scaled log-transformed row-scaled or column-scaled RPKM values (Colour Key), respectively.

Any exDNA in the bloodstream could be a target for DNase I, so during analysis we paid attention to redistribution between fragments of exDNA in L_h_, L_D_ and L_LLC_. As the first part of the analysis, we mapped the data to the reference database. The mapping rate varied from 6.95% to 8.33%, which corresponds to a number of mapped reads from 3.8 to 5.6 million. Raw read counts were normalized by the library size followed by log transformation. A heatmap with hierarchical clusterization shows the distance between technical replicates of healthy controls to be apparently less than the distance between treated and untreated mice groups (Figs 3–5).

#### GC-poor regions

Analysis of FastQC data revealed that exDNA from the L_LLC_ library was characterized by the presence of 3 × 10^6^ GC-poor sequences (0–2% of total content). DNase I treatment (L_D_) resulted in a decrease in the content of GC-poor sequences to 1.0 × 10^6^ sequences, close to the content of these sequences in the exDNA of healthy mice (0.5–1.0 × 10^6^, L_h_).

#### Tandem repeats

The most striking differences between libraries L_LLC_, L_D_ and L_h_ were observed for tandem repeats. ExDNA of mice with LLC was characterized by the presence of a bigger amount of tandem repeats in comparison with exDNA of healthy mice (Table 2). Comparative analysis of 980 types of tandem repeat presented in the *Mus musculus* genome was performed. For further analysis, 250 types of tandem repeat with representation of at least one sequence per million sequences of the library and characterized by sharp differences between the groups were selected (Table 2, Fig 3). In brief, in searching for further potent oncomarkers, number selection was done to analyze only well-presented repeat types.

**Table 2:** Abundance of 250 types of tandem repeat in DNA libraries.

	Types of tandem repeat, number	Abundance, RPM^1^			
		L_LLC_	L_D_	L_h1_	L_h2_
L_LLC_> L_h_: 204	158*	53193	43612	34469	36318
	46^#^	10221	8341	3635	3859
L_LLC_< L_h_: 46	39^¤^	17827	15726	28777	30996
	7^£^	431	504	1502	1489
Total	250	81674	68186	38104	40176

*repeat types, whose abundance was 1.5-2-fold raised during LLC development and dropped after DNAse I treatment

^#^repeat types, whose abundance was 2.5-3-fold raised during LLC development and dropped after DNAse I treatment

^**¤**^repeat types, whose abundance was 1.5-2-fold decreased during LLC development and further declining after DNAse I treatment

^£^repeat types, whose abundance was 3-fold decreased during LLC development and increased after DNAse I treatment

^1^RPM—reads per million reads in library

It was found that in LLC development, the content of exDNA was characterized by a high abundance of 204 tandem repeats in comparison with healthy mice (compare libraries L_LLC_ and L_h1_, L_h2_). The abundance of 158 types of tandem repeat in L_LLC_ was 1.5–2-fold higher, and the abundance of 46 types of repeats was 2.5–3-fold higher than in L_h1_ and L_h2_ (Table 2, Fig 3). DNase I treatment resulted in a decrease of the abundance of tandem repeats which were over-represented in untreated mice: we observed a 1.5-fold reduction in the abundance of tandem repeats in L_D_ compared with L_LLC_. In 30% of cases the decrease in repeat abundance in L_D_ reached the level of L_h1_ and L_h2_ (Table 2, Fig 3).

The abundance of 46 tandem repeat types in L_LLC_ was 1.5–2-fold lower than in L_h1_ and L_h2_ (L_LLC_ < L_h_, Table 2). Treatment with DNase I resulted in a decrease of the abundance of 39 repeat types by 10–30% and an increase of the abundance of seven repeat types by 20–50% (Table 2, Fig 3).

ExDNA of LLC-bearing mice was characterized by a high abundance of repeats belonging to 11 major subfamilies compared with healthy mice (Table 3). After treatment with DNase I we observed a decrease in abundance of these repeats by 15–25% (Table 3). It was mentioned that upon DNase I treatment, the abundance of repeats belonging to subfamilies B, IAP, LTRIS, ORR and RSINE reached the level of healthy mice (compare L_D_, L_h1_ and L_h2_, Table 3).

**Table 3:** Abundance of tandem repeats belonging to 11 major subfamilies in DNA libraries.

Subfamilies	repeats types, found in exDNA, number	Abundance, RPM^1^			
		L_LLC_	L_D_	L_h1_	L_h2_
B	19*	7548	6156	3326	3541
ERVB	11*	930	770	693	688
IAP	24*	6682	5583	3104	3272
L1	19*	27508	23523	23984	25066
LTRIS	5*	373	308	177	192
Lx	14*	3471	2861	2076	2222
	2^#^	51	46	55	59
MERV	5*	1461	1247	995	1032
MMERV	5*	812	688	451	482
	2^#^	80	72	98	103
ORR	14*	2158	1785	1237	1317
RLTR	52*	10201	7603	5877	6380
RMER	22*	895	1119	667	753
	7^¤^	1518	1934	1916	2713
RSINE	3*	201	178	132	123

*repeat types whose abundance was increased in LLC development and decreased after DNase I treatment.

^#^repeat types whose abundance was decreased in LLC development and further declined after DNase I treatment.

^¤^repeat types whose abundance was decreased in LLC development and increased after DNase I treatment.

^1^RPM—reads per million reads in library.

Completely different tendencies were observed in the case of repeats belonging to Lx, MMERV and RMER subfamilies. The abundance of 14, five and 22 repeat types (Lx, MMERV and RMER respectively) was 1.5–2-fold increased in LLC development (Table 3), and after DNase I treatment a reduction of their abundance by 30–50% was observed compared to the repeat content of healthy mice (Table 3). However, in LLC development the abundance of two, two and seven repeat types (Lx, MMERV and RMER), respectively, was lower than in those of healthy mice (Table 3) and additionally decreased after DNase I exposure (Table 3). The abundance of the majority of repeat types belonging to ERVB, L1 and RLTR subfamilies was increased in LLC development and decreased after DNase I exposure to a level of repeat content comparable with healthy mice (Table 3).

Special attention was given to 19 repeat types of B1, B2, B3 and B4 subfamilies having homology to human ALU repeats (Fig 4). In LLC development, abundance of these repeats in the exDNA content of blood was increased two-fold in comparison with healthy mice (7.5 × 10^3^ RPM in L_LLC_ vs 3.4 × 10^3^ RPM in L_h1_ and L_h2_), and DNase I treatment resulted in a reduction of 20% (to 6.1 × 10^3^ RPM in L_D_).

#### Fragments encoding tumour-specific genes

In order to find possible molecular targets of DNase I among tumour-specific genes, analysis of *Hmga2*, *Fos*, *Myc*, *Nras* and *Jun* genes known to be carcinogenesis markers [36–40] was performed (Table 4, Fig 5). In the exDNA pool of healthy mice the abundance of fragments encoding these genes was low: total abundance did not exceed 6.87–10.78 RPM (L_h1_ and L_h2_, Table 4). The major part of the fragments was found to encode *Myc* (about 50%): the abundance of these fragments was four to five times higher than fragments encoding other genes (L_h1_ and L_h2_, Table 4). In LLC development, the abundance of fragments encoding tumour-specific genes was 15-fold higher in comparison with healthy mice (compare L_LLC_, L_h1_ and L_h2_, Table 4) and 90% of all fragments belonged to *Myc*. Thus, in tumour development the abundance of *Myc*-specific fragments rose 15-fold and the abundance of *Hmga2*-, *Fos*- and *Jun*-specific fragments rose two- to three-fold in comparison with healthy mice, whereas the abundance of *Nras*-specific fragments did not change (Table 4). For DNase I treatment, a 1.5–2-fold decrease in the abundance of tumour-specific fragments of *Myc*, *Hmga2* and Jun was observed, in the case of *Hmga2* to the level of healthy mice, while the abundance of *Fos* was slightly increased (Table 4).

**Table 4:** Abundance of fragments encoding tumour-associated genes *Hmga2*, *Fos*, *Myc*, *Nras* and *Jun* in DNA libraries.

Tumor-associated gens	Abundance, RPM^1^			
	L_LLC_	L_D_	L_h1_	L_h2_
Hmga2	1.90	1.35	1.27	0.82
Fos	2.74	2.93	0.95	0.84
Myc	86.14	73.38	6.40	3.62
Nras	1.14	1.39	1.25	0.97
Jun	2.16	1.39	0.90	0.62
Total	94.08	81.00	10.78	6.87

^1^RPM—reads per million reads in library.

**Fig 6:**
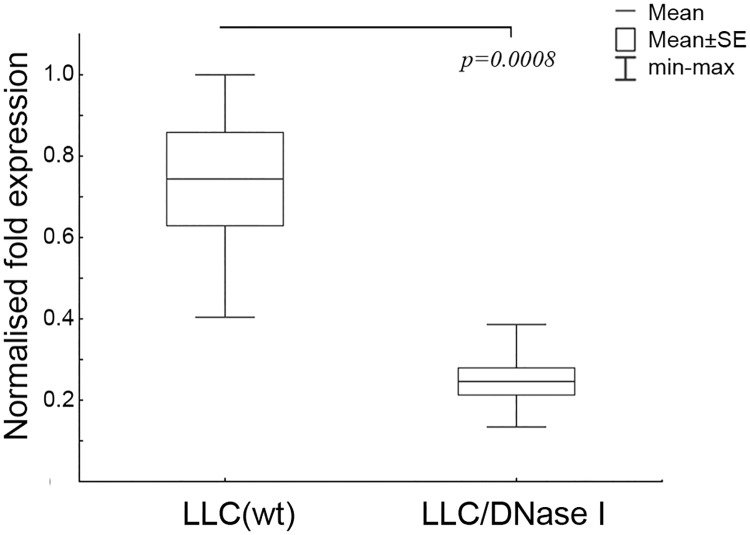
Box plots of the level of Myc-specific fragments among exDNA in the blood serum of mice with LLC treated with saline buffer and DNase I (PCR data). Starting from day 4 after the LLC transplantation, the animals received i.m. saline buffer or DNase I (0.12 mg/kg) for 10 days. LLC(wt)–mice received saline buffer, LLC/DNase I—mice received DNase I. The Myc level was normalised to β-actin level. Data were statistically analysed using one-way ANOVA with post hoc Fisher test. Data are presented as mean ± SE. Statistical significance is p < 0.0008.

Validation of the gene fragments alterations in exDNA profiles of blood serum after treatment with DNase I was performed using real-time RT-PCR technology. For validation Myc gene was chosen because of its highest abundance among other exDNA fragments encoding tumour-associated genes. Six samples from each mice group, which were used for sequencing, were investigated. The obtained data indicated an increase in the levels of Myc-specific fragments among exDNA in blood serum of mice with LLC treated with saline buffer (Fig 6). After the treatment with DNase I we observed an approximately 3-fold reduction in Myc abundance (Fig 6) that coincides with sequencing data.

## Discussion

The presence of a large amount of exDNA in the blood plasma of cancer patients was described several decades ago [41]. It was demonstrated that the major part of this exDNA had tumour origin and appeared in blood plasma as a result of necrosis and apoptosis in tumour cells [42]. Attempts to use exDNA as a source of oncomarkers were made [8,9,43,44]. On the other hand, assessment of alterations in exDNA profile as oncomarkers seems more appropriate. For a variety of tumours, a number of mutant sequences have been found in the blood of patients [10], including point mutations (SNP) [45–47], microsatellite alterations [48,49] and hypermethylation sequences [50], as well as loss of allelic variants of oncosuppressors [51,52]. Furthermore, mutant mitochondrial DNA sequences were detected [43]. The biological role of the appearance of all this tumour-derived exDNA is not well understood.

As mentioned in the introduction, Garcia-Olmo et al. put forward a hypothesis for the key role of exDNA in metastasis (genometastatic hypothesis) [13]. This hypothesis has been adopted by other authors as a model for explaining contradictions in the experimental data relating to metastasis. In 2010, Garcia-Olmo et al. showed that exDNAs originated from tumour cells are involved in malignant transformation of healthy cells. The ability of tumour-associated exDNAs to transform healthy cells was proved by detection of mutated K-ras fragments in NIH-3T3 mouse cells after the cultivation in the presence of blood plasma of colon cancer patients enriched with these fragments [53].

According to another hypothesis, exDNA forming complexes with a variety of proteins (histones, actin, peroxidase and other proteins that generate reactive oxygen species, interleukins and others) forms the genic network, also called NET, neutrophil extracellular traps [54]. NETs are used by neutrophils to catch pathogens and to migrate to pathogen locations. It was shown that tumour cells are capable of incorporating their own tumour-derived exDNA into NET, and this promotes tumour cells to metastasize [55].

In view of the possible carcinogenic role of exDNA, investigation of enzymes capable of destroying exDNA has been actively conducted [27,29,31,56]. In previous works using several tumour models, both murine and human, the antimetastatic effect of DNAse I was shown [27,29,31,56]. It should be mentioned, the antimetastatic effect was correlated with a decrease in the exDNA level and increase in the DNase I activity. However, any inhibition of primary tumour growth under DNase I treatment was not detected.

The objective of this work was to study in detail how DNase I treatment of tumour-bearing mice changes the blood serum exDNA profile after the decrease of tumour invasion potential. For this aim we used a murine LLC model which has epithelial origin and is relevant to human non-small cell lung cancer. We compared the exDNA profile of the blood of mice with LLC treated and not treated with DNase I by NGS on the ABS SOLiD^™^ 5.500 platform and found that the key components altered after the treatment. One might expect that treatment with DNase I would result in non-specific degradation of all circulating exDNA in the bloodstream; however, it led to non-random changes in the exDNA profile.

One of the questions was: are there differences in exDNA profiles between healthy mice and mice with LLC? We found that tumour development resulted in an increase in the abundance of GC-poor sequences, tandem repeats, including repeats of B1–4 subfamilies homologous to human ALU repeats and fragments encoding tumour-specific genes. An increase in the content of fragments belonging to tandem repeats and tumour-specific genes in blood exDNA was detected for different types of disease including cancer, whereas the content of GC-poor sequences in blood exDNA in the majority of studies was shown to be reduced [57–59]. The increase in the content of GC-poor sequences, which are unstable and sensitive to degradation by blood deoxyribonucleases, could be evidence of reduced DNase activity in the blood of mice with LLC [34], which correlates well with previously obtained data indicating reduced DNase activity in the blood of patients with oncological diseases [60–62]. Thus, reduction in the abundance of GC-poor sequences to the level of healthy mice observed after treatment of LLC-bearing mice with DNase I could be a consequence of increased DNase activity in blood plasma.

Upon LLC development, we observed an increase in the representation of many types of tandem repeat that refer to mobile genetic elements such as retrotransposons. Most of the repeats related to long terminal repeats, but there were also many types of LINE and SINE element, as well as other non-long terminal repeats (RMER, ORR, IAP).

A role of retrotransposition in the etiology of cancer remains open. Nevertheless due to a large amount of data concerned tumour-specific insertions of SINE and LINE elements, some hypotheses about a possible role of retrotransposons in tumour development exist [63–65]. Tumour-derived microvesicles are commonly enriched with genes, containing insertions of LINE-1 and Alu elements [66]. Some reports indicated that cancer can be initiated by retrotransposition-mediated gene inactivation by somatic insertion [67] and insertion in tumour suppressor genes [68,69]. So the insertion of full-length SVA (SINE-VNTR-Alu human retrotransposons) into intron 8 of the caspase 8 (*CASP8*) gene is associated with cutaneous basal cell carcinoma and breast cancer [70]. To date, the most commonly used markers for human carcinogenesis are ALU repeats [71–73], which were shown to be significantly over-presented in breast cancer and colorectal cancer patients [48,49,74,75]. Repeatedly, it was shown that the development of various types of cancer is accompanied by an increase in the abundance of SINE and LINE elements in circulating exDNA [74, 76–78].

We found a two-fold increase in abundance of repeats of the B-subfamily, particularly the B1-subfamily, homologous to human ALU repeats [79]. Treatment of LLC-bearing mice with DNase I caused a drop in content of repeats belonging to the B-subfamily by 20%. Taking into account the oncogenic role of these repeated elements, the observed DNase-mediated effect may give impact to the reduction in metastasis number and area. Together with the change in B1 element content in exDNA, we revealed that treatment with DNase I led to a decrease in the content of L1 elements in blood exDNA (statistically insignificant) and an essential decrease in Lx elements, concluding that Lx repeats could serve as markers of carcinogenesis, particularly for tumours of epithelial origin.

There are some evidences that DNA regions encoding tumour-associated genes are duplicated in tumour cells of different histogenesis, for example, c-Myc was shown to be duplicated in pediatric osteosarcoma [80]. The abundance of tumour-associated gene fragments in blood under tumour progression is a result of necrosis and apoptosis of tumour cells from primary tumour node.

We found that upon LLC development, enhanced levels of fragments encoding tumour-specific genes *Hmga2*, *Fos*, *Myc* and *Jun* were detected in the blood of tumour-bearing mice, and after DNase I treatment levels of all fragments except for *Fos*-specific ones were reduced. As cancer markers, mutant forms of *K-Ras*, *p53* and *N-Myc* are widely used [1,2,10,11,12,45]. Taking into account the abundance of *Jun*- and *Fos*-specific fragments in exDNA content in the blood of LLC-bearing mice and the 2.5–3-fold increased level of these fragments in comparison with healthy mice, these genes can be proposed as possible markers of malignant diseases, particularly for detection of lung cancer. So, taking into account the hypothesis of Garcia-Olmo, we assume that there is a connection between the decrease in the number of tumour-associated gene sequences in the blood and a decrease in the level of metastasis.

In spite of essential success in studies of the antimetastatic potential of DNase I, the mechanism of action and possible targets of the enzyme have not yet been clarified. There are two possible mechanisms of DNase I antitumour effect. According to one of them, DNase I via binding with different peptides (histones, actins, peroxidases and other peptides generating active oxygen forms) may degrade exDNA which participates in the formation of NET. Tumour cells are capable of using NET and integrate their own tumour-derived exDNA, facilitating malignant spreading. According to the second mechanism, DNase I is capable of cleaving circulating tumour-specific exDNA which participates in malignant transformation of normal cells through horizontal transfer.

## Conclusion

To date, the problem of finding new low-invasion methods for detecting and monitoring the tumour process is one of the most urgent. In this study, we conducted a detailed study of the influence of DNase I on the exDNA profile in the blood serum of mice with LLC and proved the antimetastatic effect of the enzyme. The most important result was finding a spectrum of new possible LLC oncomarkers, which can be regarded as oncomarkers of non-small cell lung cancer. The major differences were observed for the following types of sequence: GC-poor sequences, tandem repeats (sequences with high stability) and fragments relevant to tumour-specific genes (*Hmga2*, *Fos*, *Myc* and *Jun*). The most important result obtained in the work is that DNase I treatment caused a decrease of the level of B-subfamily repeats having homology to human ALU repeats to the level of healthy animals, and we also showed that the antimetastatic effect of DNase could be due to its influence on the profile of exDNA.

## Supporting information

S1 Fig Typical histotopograms of lung lobes in the groups of LLC-bearing mice treated with saline buffer (A panel) and treated with DNase I at the dose of 0.12 mg/kg (B panel). Haematoxylin and eosin staining. Arrows indicate large metastases. Bar corresponds to 5 mm.
(DOCX)
