# Supplementary material for: Alteration of the exDNA profile in blood serum of LLC-bearing mice under the decrease of tumour invasion potential by bovine pancreatic DNase I treatment
Source: PLoS One. 2017 Feb 21;12(2):e0171988. doi: 10.1371/journal.pone.0171988 (PMC5319761; doi:10.1371/journal.pone.0171988)
Supplement: S1 Fig — Haematoxylin and eosin staining. Arrows indicate large metastases. Bar corresponds to 5 mm. (DOCX) [file pone.0171988.s001.docx]

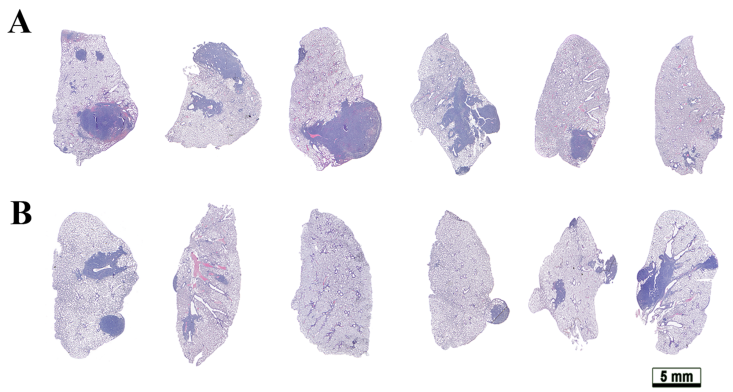


**Fig. S1.** Typical histotopograms of lung lobes in the groups of LLC-bearing mice treated with saline buffer (A panel) and treated with DNase I at the dose of 0.12 mg/kg (B panel). Haematoxylin and eosin staining. Bar corresponds to 5 mm.
